# Antibacterial efficacy and compressive strength of modified glass ionomer cements

**DOI:** 10.6026/973206300213135

**Published:** 2025-09-30

**Authors:** Kalyana Ponangi, Satish Venkata Sarvepalli, Anil Kumar enkeshwarlu James, Sandeep Pasumarthy, Srisha Neshangi, Sruthi Katamneni

**Affiliations:** 1 Noble Community Clinics, Wautoma, Wisconsin, USA; 2 Department of Conservative Dentistry, Navodaya Dental College, Karnataka, India; 3 Jefferson Dental and Orthodontics, San Antonio, Texas, USA; 4 Affordable Dentures and Implants, Danville, Virginia, USA; 5 Prosmiles Dental, Plano, Texas, USA

**Keywords:** Glass ionomer cement, antibacterial efficacy, compressive strength

## Abstract

Incorporation of antibacterial agents into restorative materials often compromises the physical properties of glass ionomer cement (GIC). Therefore, it is of interest to evaluate the antibacterial efficacy and compressive strength of FUJI IX GIC modified with 2% chlorhexidine, 1% cetrimide, 2.5% triclosan, or 1.5% antibiotics (metronidazole, minocycline and ciprofloxacin), added to the powder at a powder-liquid ratio of 3.6/1. Compressive strength was assessed using 50 cylindrical specimens (4 mm diameter, 6 mm height, 10 per group) tested on a Universal Testing Machine, while antibacterial efficacy was evaluated via agar diffusion on 50 disc-shaped specimens (10 mm diameter, 2 mm thickness, 10 per group) measuring inhibition zones on BHI agar. Statistical analysis involved ANOVA followed by Dunnett's t-test, with p < 0.05 considered significant; all modified GICs exhibited antibacterial activity but showed significantly reduced compressive strength compared to the control. The antibiotic-modified GIC demonstrated superior antibacterial efficacy and compressive strength among the experimental groups, though the control group had the highest overall compressive strength.

## Background:

A new method for treating dental caries, known as Atraumatic Restorative Treatment (ART), was presented at the World Health Organization headquarters on World Health Day in 1994 [1]. This minimally invasive technique, requiring no drill, running water, or electricity, has been a boon for patients in rural areas with limited access to dental care. ART combines preventive and restorative procedures to arrest caries progression and alter the oral microbiota, using hand instruments to remove decalcified tooth tissue and adhesive restorative materials to restore teeth [2]. Developed in Tanzania in the mid-1980s, ART emphasizes minimal intervention and maximal prevention to preserve sound tooth tissues [2]. The World Health Organization recognized and promoted ART globally in 1994 during the International Association for Dental Research (IADR) meeting, addressing oral care needs in economically less developed regions [1]. ART has been recommended for managing small, occlusal carious lesions in primary and permanent teeth and is gaining acceptance in developed countries for treating caries in children, mentally challenged patients and geriatric populations [3]. ART uses hand instruments for caries removal, which may leave residual carious dentin. Studies show hand excavation is less effective than rotary burs and residual bacteria may lead to secondary caries, causing restoration failure [4]. Conventional glass ionomer cement (GIC) is the preferred restorative material for ART due to its self-curing and caries-inhibiting properties [5]. GIC exhibits anticariogenic effects, potentially preventing secondary caries even in high-risk conditions [6]. However, residual bacteria under GIC restorations remain viable for up to two years [7], suggesting that GIC's caries-inhibiting effect may not fully arrest caries progression. Combining antibacterial agents with GIC could enhance therapeutic outcomes [8]. GIC with antimicrobial agents reduces viable bacteria while preserving dentin and pulpal vitality [9]. However, these agents often compromise GIC's physical properties [10, 11 and 12]. Previous studies evaluated chlorhexidine (2%), cetrimide (1%) [13], triclosan (2.5%) [14] and antibiotics (metronidazole, minocycline, ciprofloxacin) (1.5%) [15] individually with GIC, concluding that these experimental GICs demonstrated adequate antibacterial efficacy without significantly affecting physical properties. However, no study has compared all these agents together. Therefore, it is of interest to evaluate the antibacterial efficacy and compressive strength of glass ionomer cements combined with chlorhexidine (2%), cetrimide (1%), triclosan (2.5%) and antibiotics (metronidazole, minocycline, ciprofloxacin) (1.5%).

## Materials and Methods:

## Source of materials:

A conventional powder and liquid FUJI IX Glass Ionomer Cement (GC, Tokyo, Japan) was used along with antimicrobial agents, including:

[1] Chlorhexidine (2%) (Smaart Pharmaceuticals, Jalgaon, Maharashtra, India)

[2] Cetrimide (1%) (Smaart Pharmaceuticals, Jalgaon, Maharashtra, India)

[3] Triclosan (2.5%) (V2 Chempharma Pvt. Ltd., Hyderabad, Telangana, India)

[4] Antibiotics (ciprofloxacin, minocycline and metronidazole) (1.5%) (Aurobindo Pharmaceuticals, Hyderabad, Telangana, India)

## Methodology:

## Sample:

Conventional restorative Glass Ionomer Cement (FUJI IX, GC, Tokyo, Japan) was used as the control. Five groups (one control and four experimental) were assessed, with 10 specimens per group for both antibacterial efficacy and compressive strength, totaling 100 specimens. The groups are as follows:

## Specimen preparation:

The dimensions of the specimens varied depending on the parameter tested, as shown below:

## Preparation of antibacterial cements:

A conventional restorative Glass Ionomer Cement (FUJI IX, GC, Tokyo, Japan) was used as the control. In experimental groups, FUJI IX powder was mixed with antimicrobial agents: chlorhexidine (2%), cetrimide (1%), triclosan (2.5%) and antibiotics (ciprofloxacin, minocycline, metronidazole) (1.5%), using a powder-liquid ratio of 3.6:1 as depicted in Table 1 (see PDF).

## Antimicrobial activity screening tests:

The antimicrobial efficacy was evaluated against pure strains of Streptococcus mutans (MTCC 890) from the Microbial Type Culture Collection (MTCC), Chandigarh, using agar diffusion tests. Strains stored at -20°C were cultured on blood agar (Hi Media Pvt. Ltd., Mumbai, Maharashtra, India) at 37°C for 24 hours in 5% CO_2_. Single colonies were transferred to Brain Heart Infusion (BHI) broth (Hi Media Pvt. Ltd., Mumbai, Maharashtra, India) and incubated at 37°C for 24 hours. Suspensions of 1.5x10^8^ organisms/ml (McFarland 0.5 turbidity standard) were prepared in Phosphate Buffered Solution and flood-inoculated onto BHI agar plates. The plates were air-dried at 37°C for 15 minutes before placing set disc-shaped specimens (10 mm diameter, 2 mm thick; Table 2 - see PDF), prepared with a powder-liquid ratio of 3.6:1. After setting at room temperature for 30 minutes, specimens were placed on BHI agar plates. After incubation at 37°C for 48 hours, inhibition zones were measured by subtracting 10 mm (well diameter) from the average zone diameter for each specimen and control.

## Compressive strength testing:

Compressive strength testing was conducted per EN-ISO 9917 (Dental Water-based Cements, 1991). Cylindrically shaped specimens (6 mm height, 4 mm diameter; Table 2 - see PDF) were prepared using Teflon molds with a powder-liquid ratio of 3.6:1. Specimens were stored at 37 ± 1°C in 100% humidity for 60 minutes after mixing, then in distilled water for 24 hours and 7 days. Compressive strength was measured using a Universal Testing Machine (Dak System Inc., Mumbai, Maharashtra, India) at a crosshead speed of 1 mm/min. Testing was conducted at the Micro, Small and Medium Enterprises (MSME), Central Institute of Tool Design, Hyderabad.

## Plan for statistical analysis:

Descriptive statistics (mean, standard deviation, percentage) were used. Comparisons between groups were performed using ANOVA for multiple group comparisons, followed by Dunnett's t-test for comparison with the control group. A p-value less than 0.05 were considered significant.

## Results:

All groups, including the control, demonstrated significant antimicrobial efficacy. Group V (1.5% antibiotics: ciprofloxacin, metronidazole, minocycline) exhibited the highest antimicrobial efficacy. The order of antimicrobial efficacy is: Group V > Group III > Group IV > Group II > Group I. All the inhibition zones are seen in Figures 1-5 (see PDF). The mean and standard deviation values are presented in Table 3 (see PDF). All experimental groups exhibited lower compressive strength compared to the control group (Group I: FUJI IX Glass Ionomer Cement). Group V (1.5% antibiotics) showed the least reduction in compressive strength among the experimental groups. The order of compressive strength results is: Group I > Group V > Group III > Group IV > Group II. The mean and standard deviation values are presented in Table 4 (see PDF).

## Discussion:

Incorporation of chlorhexidine dihydrochloride and diacetate into GICs enhances antimicrobial effects without significantly compromising physical properties [16, 17, 18- 19]. Takahashi *et al.* [10] used High Performance Liquid Chromatography (HPLC) to show significant chlorhexidine (CHX) release from experimental GIC formulations, concluding that 1% CHX diacetate was optimal for FUJI IX. Based on Emilson *et al.* [20], this study selected 2% CHX, known for its antibacterial activity against caries-associated bacteria, incorporated as a powder into FUJI IX. Higher concentrations of CHX compromised restorative material properties, justifying the 2% CHX selection. This study found that 2% CHX increased antibacterial properties against Streptococcus mutans compared to conventional GIC (mean inhibition zone: 4.90 ± 0.22 mm vs. 2.75 ± 0.36 mm, p < 0.01), but its efficacy was lower than other agents tested. Additionally, 2% CHX caused a greater reduction in compressive strength (mean: 101.18 ± 1.62 MPa) compared to other agents, with a mean difference of 64.76 MPa from the control. Cationic disinfectants, such as cetrimide (CT), cetylpyridinium chloride (CPC) and benzalkonium chloride (BC), have been studied for their antibacterial effects [17, 11]. This study used 1% cetrimide in FUJI IX GIC, as Deepalakshmi *et al.* [13] found this concentration optimal for antibacterial effects without compromising physical properties. Our results confirmed significant antimicrobial activity (mean inhibition zone: 6.05 ± 0.21 mm, p < 0.01) and maintained physical properties (mean compressive strength: 141.49 ± 1.35 MPa), with cetrimide showing higher antibacterial efficacy and compressive strength than chlorhexidine (2%) and triclosan (2.5%) but lower than antibiotics (1.5%). Sato *et al.* [21] found the ternary antibiotic combination effective in sterilizing carious lesions, necrotic pulps and infected root dentin in deciduous teeth, both *in vitro* and *in vivo*. In this study, the antibiotic mixture (1.5%) demonstrated the highest antibacterial efficacy (mean inhibition zone: 10.70 ± 0.43 mm, p < 0.01) and the least reduction in compressive strength (mean: 149.29 ± 2.56 MPa) among experimental groups. Triclosan, a broad-spectrum antimicrobial agent, was incorporated into FUJI IX GIC at 2.5% concentration, as investigated by Sainulabdeen *et al.* [14]. This study found that triclosan significantly enhanced antibacterial efficacy against Streptococcus mutans (mean inhibition zone: 5.27 ± 0.31 mm, p < 0.01) compared to the control group, though its efficacy was lower than cetrimide and antibiotics but higher than chlorhexidine. The incorporation of triclosan, however, resulted in a notable reduction in compressive strength (mean: 130.98 ± 1.44 MPa), with a mean difference of 34.96 MPa from the control, placing it between cetrimide and chlorhexidine in terms of physical property impact. Somani *et al.* [22] reported that triclosan-incorporated GIC exhibited adequate antibacterial properties but increased microleakage, suggesting potential limitations in clinical sealing performance. The 2.5% concentration used in this study aligns with prior research indicating effective antibacterial activity, but the trade-off in compressive strength highlights the need for careful consideration of triclosan's concentration to balance antimicrobial benefits and mechanical integrity.

## Conclusion:

All experimental Glass Ionomer Cements (GICs) demonstrated antibacterial efficacy, but the incorporation of antibacterial agents significantly reduced compressive strength. Experimental GIC containing antibiotics (1.5% metronidazole, ciprofloxacin and minocycline) exhibited greater antibacterial efficacy and compressive strength compared to other experimental groups. The highest compressive strength was observed in the control group (FUJI IX).
